# Crosstalk between neuroinflammation and ferroptosis: Implications for Parkinson’s disease progression

**DOI:** 10.3389/fphar.2025.1528538

**Published:** 2025-03-13

**Authors:** Xiangyu Guo, Ran Wei, Xunzhe Yin, Ge Yang

**Affiliations:** ^1^ College of Traditional Chinese Medicine, Changchun University of Chinese Medicine, Changchun, China; ^2^ Cardiovascular Surgery Department, Second Hospital of Jilin University, Changchun, China; ^3^ Center for Theoretical Interdisciplinary Sciences, Wenzhou Institute, University of Chinese Academy of Sciences, Wenzhou, China

**Keywords:** Parkinson’s disease, neuroinflammation, ferroptosis, natural products, microglia, astrocytes

## Abstract

Parkinson’s disease (PD) is a common neurodegenerative disorder characterized by the degeneration of dopaminergic neurons and the aggregation of α-synuclein. Neuroinflammation is triggered by the activation of microglia and astrocytes, which release pro-inflammatory factors that exacerbate neuronal damage. This inflammatory state also disrupts iron homeostasis, leading to the occurrence of ferroptosis. Ferroptosis is characterized by lipid peroxidation of cell membranes and iron overload. Abnormal accumulation of iron in the brain increases oxidative stress and lipid peroxidation, further aggravating neuroinflammation and damage to dopaminergic neurons. Natural products have garnered attention for their antioxidant, anti-inflammatory, and neuroprotective properties, with many plant extracts showing promising therapeutic potential in PD research. This study further investigates the potential therapeutic roles of various natural products in regulating neuroinflammation and ferroptosis. The results suggest that natural products have significant therapeutic potential in modulating the interaction between neuroinflammation and ferroptosis, making them potential treatments for PD. Future research should further validate the safety and efficacy of these natural compounds in clinical applications to develop novel therapeutic strategies for PD.

## 1 Introduction

The prevalence of Parkinson’s disease (PD), the second most prevalent neurological illness, rises with age and can reach 1% in people over 60 (Thapa et al., 2022). The symptoms of PD are classified into motor and non-motor symptoms (Jankovic and Tan, 2020; Reich and Savitt, 2019). Motor symptoms are also the most recognizable features, primarily including tremors, stiffness, motor dysfunction, and abnormal gait (Bloem et al., 2021). Surveys show that dysphagia affects more than 80% of PD patients at some point during the illness (Kalf et al., 2012). Dysphagia lowers quality of life, makes it more difficult to take medications, and increases the risk of aspiration pneumonia and malnutrition—two major causes of death in PD (Lin et al., 2012). Non-motor symptoms of PD onset insidiously, and modern research conceptualizes them as a complex neuropsychiatric disorder. Typical non-motor symptoms include emotional aspects (depression and anxiety), perceptual and cognitive changes (psychosis), and motivational issues (impulse control disorders and apathy) (Weintraub et al., 2022). Depression is one of the most commonly reported non-motor symptoms in PD, with 40%–50% of patients experiencing clinically significant depressive symptoms (Marsh, 2013). Additionally, the probability of PD patients developing dementia is as high as 30% (Hanagasi et al., 2017). The high occurrence of non-motor symptoms in PD patients significantly increases the clinical complexity of the disease, directly resulting in notable declines in cognitive function and quality of life. The combination of motor and non-motor symptoms in PD patients highlights the complexity and multifaceted nature of this neurodegenerative disorder, further exacerbating the overall burden of the disease, impacting not only the quality of life of patients but also increasing the challenges faced by caregivers.

The pathological features of PD are the death of dopaminergic neurons (DAn) and the intracellular deposition of α-synuclein (α-syn) (Kalia and Lang, 2015). The immune system experiences senescence as we age, which causes the innate and adaptive immune systems’ capacities to gradually deteriorate and reduce our capacity to control inflammation (Tansey et al., 2022). Neuroinflammation is characterized by the release of inflammatory mediators from activated microglia and astrocytes, such as cytokines IL-1β, IL-6, and TNF-α (Calabrese et al., 2018). These inflammatory factors not only exacerbate neuronal damage but also trigger dysregulation of iron homeostasis, promoting the occurrence of ferroptosis (Cheng et al., 2021). Lipid peroxidation and iron ion overload cause cell membrane damage in ferroptosis, an iron-dependent kind of cell death (Jiang et al., 2021). The excessive accumulation of iron ions in the brain increases the generation of free radicals and lipid peroxidation, exacerbating neuroinflammation and further damaging Dan (Yan et al., 2021; Ko et al., 2021). The accumulation of α-syn is associated with increased inflammatory gliosis and ROS production in PD brains (Rocha et al., 2018). Moreover, studies have reported that α-syn can enhance the sensitivity of DAn to ferroptosis (Mahoney-Sanchez et al., 2022). The interaction between neuroinflammation and ferroptosis in PD may be a crucial mechanism leading to dopaminergic neuron damage. Therefore, a deeper understanding of this mechanism can aid in developing targeted therapeutic strategies, slowing the progression of PD, and improving patients’ quality of life.

## 2 Neuroinflammation

**FIGURE 1:**
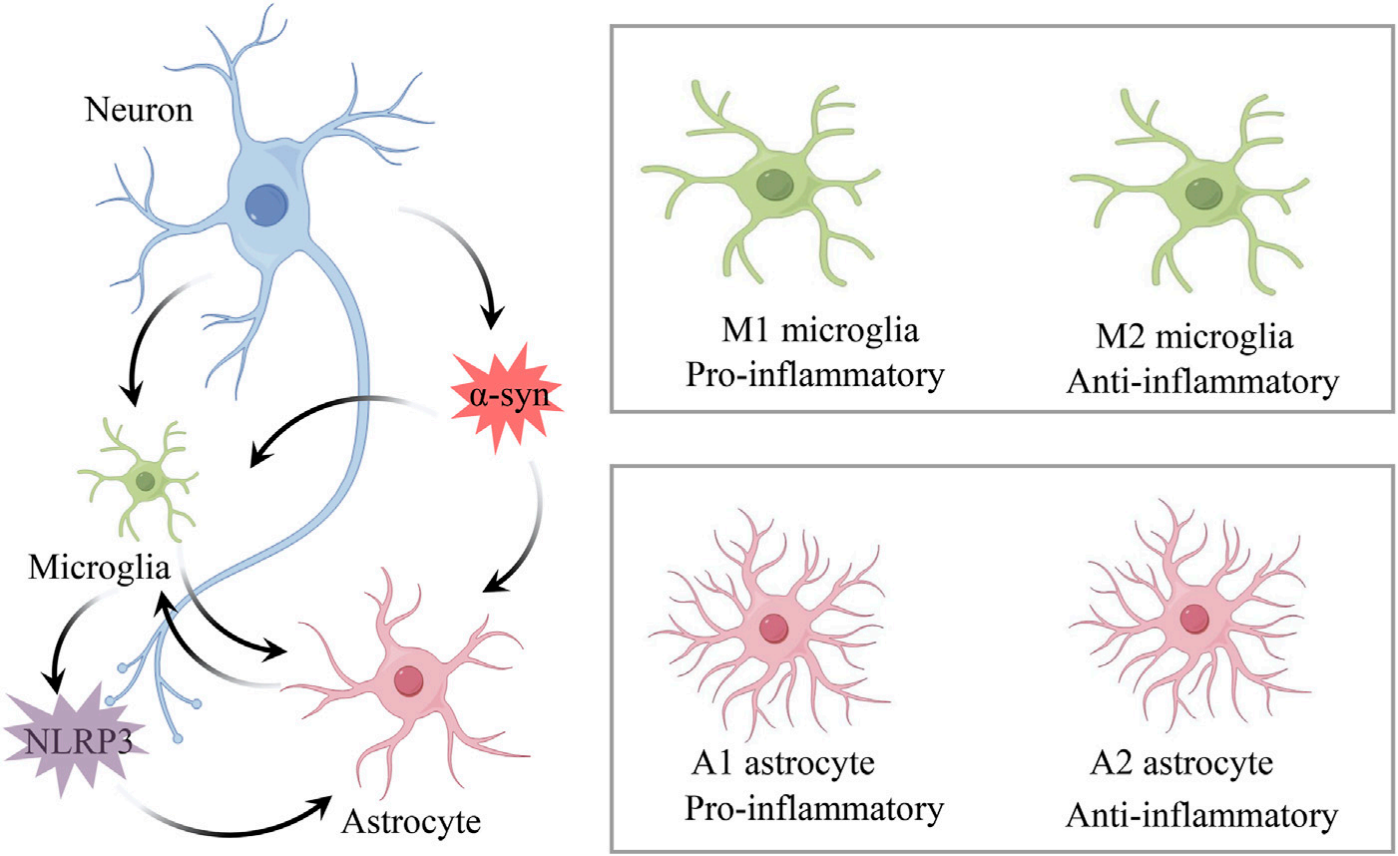
Mechanisms of neuroinflammation in PD. Microglia and astrocytes are key immune cells in CNS, playing crucial roles in maintaining CNS homeostasis and regulating the development of neuroinflammation. Under neuronal degeneration and α-syn stimulation, microglia differentiate into pro-inflammatory M1 and anti-inflammatory M2 phenotypes, while reactive astrocytes differentiate into pro-inflammatory A1 and anti-inflammatory A2 phenotypes. α-syn aggregation induces microglial activation of the NLRP3 inflammasome, which further regulates the activation of A1 astrocytes.

When inflammation occurs in the central nervous system (CNS), it is called neuroinflammation. Neuroinflammation appears to be part of the pathophysiological mechanisms of PD, but its exact pathogenic mechanism has not yet been elucidated. Several researchers have found that post-mortem human brain tissue samples from PD subjects exhibit a greater number of activated microglia in the substantia nigra (SN), indicating that inflammation is involved in PD (Croisier et al., 2005; Desai Bradaric et al., 2012; Jyothi et al., 2015). Subsequent genome-wide association studies have confirmed this hypothesis by showing increased expression of specific genes encoding HLA-DR and reduced DNA methylation (Nalls et al., 2011). Early acute neuroinflammation in the brain often exerts neuroprotective functions, facilitating neuronal repair and maintaining homeostasis, whereas prolonged and excessive activation of neuroinflammation leads to the excessive release of inflammatory mediators, causing neuronal damage and degeneration (Pintado et al., 2012; Corwin et al., 2018). Microglia and astrocytes, as important immune cells in CNS, perform functions which include recognizing danger signals, producing inflammatory mediators, and clearing microorganisms (de Araújo Boleti et al., 2020; Li et al., 2024). They play key roles in maintaining CNS homeostasis and regulating the development of neuroinflammation (Figure 1).

### 2.1 Microglia

Under physiological and pathological conditions, microglia play a key role in maintaining homeostasis and defense in CNS (Hanisch, 2002). They promote neuron survival through the phagocytosis of apoptotic cells in the subgranular zone, actively engage in synaptic pruning, and support normal brain development (Ziv et al., 2006). Research indicates that microglia act as biological sensors to regulate neuronal activity, and any changes in DAn, such as α-syn aggregates, apoptotic cells, and neurotransmitter release, may trigger a response (Salter and Stevens, 2017). This is consistent with reports showing that activated microglia in PD patients closely interact with neurons presenting pathological α-syn aggregates (Duffy et al., 2018). Scheiblich et al. found that microglia create functional networks through F-actin-dependent cell-cell connections, transferring α-syn from overloaded cells to neighboring cells, thereby alleviating the individual burden (Scheiblich et al., 2021). However, while early microglial responses support neuron survival, prolonged and excessive activation exacerbates neuroinflammation and produces cytokines that promote inflammation, including IL-1β, IL-6, and TNF-α (Mogi et al., 1994a; Mogi et al., 1994b). Aging leads to a decline in the ability of microglia and monocytes to phagocytize α-syn. This diminished capacity is accompanied by an increased secretion of pro-inflammatory cytokine TNFα, which may accelerate protein aggregation and inflammation, exacerbating the progression of neurodegenerative diseases. Additionally, the reduced efficiency of microglia in handling α-syn is compounded by inflammatory molecules released from degenerating neurons, further amplifying neuroinflammation and creating a deleterious feedforward loop (Bliederhaeuser et al., 2016). Furthermore, degenerating neurons release intrinsic pro-inflammatory active components, triggering a harmful feedforward loop that elicits microglial responses, leading to a progressively worsening immune reaction (Fuzzati-Armentero et al., 2019).

Under normal conditions, microglia are in a homeostatic state, extensively contacting other cells and blood vessels to monitor environmental changes (Salvi et al., 2017). Upon pathological stimulation, they transform into pro-inflammatory M1 and anti-inflammatory M2 phenotypes (Zeng et al., 2024). In PD, the M1 phenotype may be induced by α-syn aggregates, signaling through Toll-like receptors (TLR), releasing cytokines and neurotoxic molecules that promote cytotoxic responses, leading to persistent neuroinflammation (Li et al., 2021a). Depletion of M1 microglia can control inflammation and reduce the loss and defects of tyrosine hydroxylase (TH)-positive nigral neurons induced by MPTP (Yan et al., 2018). M2 microglia are subdivided into M2a, M2b, and M2c subtypes (Chhor et al., 2013). M2a releases neurotrophic factors and IL-10, facilitating repair and regeneration. M2b secretes IL-1β, IL-6, and TNF-β, in addition to the anti-inflammatory cytokines IL-10 and IL-12. M2c mediates anti-inflammatory effects through the release of IL-10 and TGF-β. NF-κB is a key factor in various autoimmune diseases and inflammation. Many regulators alleviate inflammatory responses by switching microglia from the M1 to the M2 phenotype through inhibition of NF-κB (Liu et al., 2020a; Zhang et al., 2019). Therefore, regulating microglial polarization is of significant importance for the treatment of PD. In addition, the M1/M2 phenotype classification is actually a simplified concept that was originally developed based on *in vitro* stimulation techniques. In recent years, with advancements in technology, the phenotypic diversity of microglia has proven to be more complex than previously thought. Single-cell genomic studies have revealed the existence of new microglial subpopulations and cell-type-specific gene sets (Martirosyan et al., 2024).

### 2.2 Astrocytes

The most prevalent cells in the brain are called astrocytes (Zhou et al., 2019). “Reactive astrogliosis,” which is defined by changed gene expression, hypertrophy, and proliferation, is a condition where astrocytes undergo molecular, cellular, and functional alterations as a result of CNS injury or disease (Sofroniew and Vinters, 2010). Reactive astrocytes are classified into two phenotypes, A1 and A2, based on differences in gene expression (Li et al., 2019). In PD, astrocytes enhance the inflammatory response by interacting with microglia and secreting IL-1α, TNF-α, and Complement component 1q to activate the pro-inflammatory A1 phenotype (Kwon and Koh, 2020). In contrast, A2 astrocytes upregulate neurotrophic factors such as glial cell line-derived neurotrophic factor (GDNF), exerting neuroprotective effects, promoting neuronal survival, and tissue repair (Fan and Huo, 2021). In animal models of PD, astrocytes reduce dopaminergic neuron death by activating the antioxidant defense system (Wei et al., 2020). Additionally, astrocytes have specialized adaptive mechanisms. When neurons are damaged, astrocytes optimize their metabolic functions, producing lactate, glutamate, and ketone bodies to provide necessary energy and maintain extracellular homeostasis (Herrera Moro Chao et al., 2022). However, astrocytes may also undergo morphological changes and increase in number. Therefore, these adaptive mechanisms may also lead to astrocytes acquiring a pro-inflammatory, senescence-associated phenotype (Turnquist et al., 2019). As astrocytes age, their ability to maintain neuronal environmental homeostasis and counteract inflammatory responses gradually diminishes, further exacerbating neuronal damage and disease progression. This dual role makes astrocytes a critical target for research and treatment in PD. In recent years, with the emergence of novel research techniques such as single-nucleus transcriptome analysis, the existence of astrocyte phenotypes beyond the A1/A2 phenotypes has been revealed. Various subtypes of astrocytes with distinct gene expression profiles have been identified (Zhu et al., 2024). Therefore, a comprehensive and thorough understanding of astrocyte heterogeneity, along with the identification of disease-specific subtypes and phenotypes, is crucial for developing therapeutic strategies targeting reactive astrocytes in PD.

### 2.3 NLRP3 inflammasome

The NLR Family Pyrin Domain Containing 3 (NLRP3) inflammasome is a central hub of immune responses, mediating the secretion of pro-inflammatory cytokines. It plays a crucial role in regulating inflammatory responses through interactions with other cellular compartments (Swanson et al., 2019). NLRP3 consists of three components: pattern recognition receptors, the signaling adapter ASC, and the caspase-1 protease (Latz et al., 2013). Different pathogenic factors or when IL-1β is secreted by microglia and sensed by NLRs, activate ASC and recruit pro-caspase-1, leading to the synthesis or release of IL-1β and IL-18 (Heneka et al., 2018). NLRP3 inflammasome activation is a key driving factor in the pathological changes of PD. Reports indicate widespread activation of inflammasomes in the brains of PD patients observed during autopsies, with elevated expression of NLRP3 at both protein and mRNA levels (von Herrmann et al., 2018). Histological studies of PD patients indicate that NLRP3 expression is significantly higher in the SN, with more than half of the cells being NLRP3 positive (von Herrmann et al., 2018). More importantly, the activation of microglia induced by the aggregation of α-syn with TLRs may be a critical step in the activation of the NLRP3 inflammasome. In NLRP3 knockout PD mouse models, reduced microglial activation, IL-1β production, and caspase-1 activation have been observed (Ou et al., 2021). It has been reported that the NLRP3 inflammasome in microglia can regulate the activation of A1 astrocytes, thereby modulating neurotoxic functions (Li et al., 2022b). Therefore, therapeutic strategies targeting NLRP3 and its downstream signaling pathways may help modulate the interactions between microglia and astrocytes, thereby alleviating the progression of PD.

## 3 Ferroptosis in PD

**FIGURE 2:**
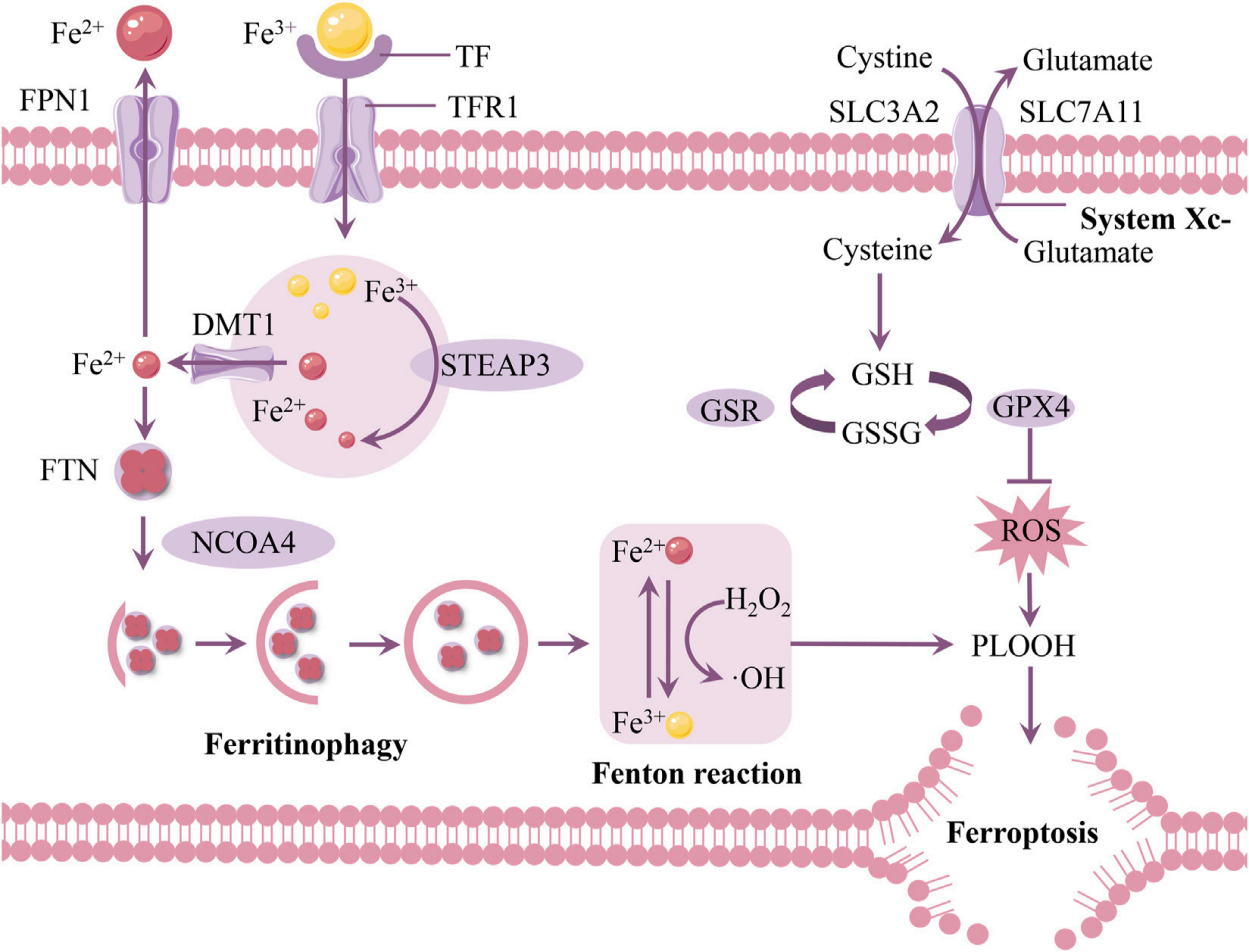
Mechanisms of ferroptosis in PD. The depletion of intracellular GSH, the reduced activity of GPX4, and excess iron ions generating large amounts of ROS via the Fenton reaction promote the accumulation of lipid peroxides and induce ferroptosis. Abbreviations: FPN1, ferroportin 1; TF, transferrin; TFR1, transferrin receptor 1; FTN, ferritin; DMT1, divalent metal transporter 1; STEAP3, six-transmembrane epithelial antigen of the prostate 3; ROS, reactive oxygen species; GPX4, glutathione peroxidase 4; GSH, glutathione; NCOA4, nuclear receptor coactivator 4.

Iron buildup in the SN of PD patients is closely linked to the loss of DAn, according to pathological and brain imaging investigations (Depierreux et al., 2021; Biondetti et al., 2021). This suggests that iron homeostasis imbalance may be a significant component in neuronal death in PD. The essence of ferroptosis lies in abnormal iron-related metabolism within cells, leading to intracellular redox imbalance, causing lipid peroxide metabolism disorder, and subsequently inducing cell death (Tang et al., 2021; Wang et al., 2024) (Figure 2). Although current evidence suggests that ferroptosis play important roles in neuronal death in PD, the mechanisms leading to neurodegeneration remain unclear. There is an urgent need to strengthen research on the mechanisms of ferroptosis in PD.

### 3.1 Iron metabolism

Iron mostly attaches to transferrin (TF) in bodily fluids like cerebrospinal fluid and is internalized into cells by clathrin-mediated endocytosis that is dependent on transferrin receptor 1 (TFR1) (Belaidi and Bush, 2016). Iron is liberated from TF once it enters the body because of its acidic environment, and the metalloreductase STEAP3 converts the bonded insoluble Fe^3+^ to the soluble ferrous (Fe^2+^) form (Knutson, 2007). Divalent metal transporter 1 (DMT1) releases iron into the cytosol, and TF and TFR1 are recycled back into the membrane for subsequent usage (Wang et al., 2022c).

When iron is taken up by cells, it binds to ferritin (FTN) for storage (Arosio et al., 2017). Ferritin is a complex composed of 24 subunits, including ferritin heavy chain and ferritin light chain, and it can store up to 4,500 iron ions (Zhang et al., 2021). If intracellular iron homeostasis is disrupted, excess free iron accumulates in cells, triggering the Fenton reaction, leading to excessive production of ROS, which damages the cell’s antioxidant system and ultimately causes ferroptosis (Dixon and Stockwell, 2014). It is noteworthy that ferritinophagy mediated by nuclear receptor coactivator 4 (NCOA4) releases Fe^2+^, leading to an increase in intracellular iron levels (Santana-Codina et al., 2021).

Ferroportin 1 (FPN1) is currently the only known iron export channel on the cell membrane. Reduced FPN1 expression and the subsequent decrease in iron excretion from brain cells are thought to contribute to elevated iron levels in the brain in PD (Qian et al., 2023). FPN1 regulation of iron level changes primarily involves two mechanisms: iron regulatory proteins (IRPs) and hepcidin. Post-translationally, iron regulatory proteins (IRP1 and IRP2) can control several iron metabolism genes, including FTH and TFR1, to maintain the stability of the labile iron pool (Wang et al., 2022a). Hepcidin significantly reduces brain iron content by regulating the expression of FPN1 in the blood-brain barrier (BBB) as well as in neurons and astrocytes (Qian and Ke, 2020).

### 3.2 Amino acid metabolism

As early as the 1950s and 1960s, Harry Eagle observed a form of cell death similar to ferroptosis, which he described as amino acid-dependent cell death (Eagle, 1955). With the definition of ferroptosis, increasing research has demonstrated that amino acid metabolism is closely related to the regulation of ferroptosis (Angeli et al., 2017). At the molecular level, the availability of cysteine, the synthesis of glutathione (GSH), and the proper function of glutathione peroxidase 4 (GPX4) are central to regulating ferroptosis.

Cystine/glutamate transporter (System Xc-) is an amino acid antiporter that mediates the exchange of extracellular cystine and intracellular glutamtate across the plasma membrane (Tu et al., 2021; Wang et al., 2020a). System Xc-is composed of two subunits: the light chain subunit solute carrier family 7 member 11 (SLC7A11) and the heavy chain subunit solute carrier family 3 member 2 (SLC3A2). (Fu et al., 2023; Liu et al., 2020b). These subunits are linked by an extracellular covalent disulfide bond and perform distinct functions. SLC3A2 primarily maintains the structural stability of System Xc- (Lin et al., 2020; Liu et al., 2021a). A crucial regulatory protein of ferroptosis, SLC7A11 acts as a transmembrane transporter that exchanges extracellular cystine for intracellular glutamate at a 1:1 ratio. (Koppula et al., 2021). This exchange supports glutathione synthesis, protects cells from oxidative stress, and maintains cellular redox balance (Hong et al., 2021).

One of the factors limiting the rate of GSH synthesis is cysteine (Wang et al., 2020a). GSH exists in the body as reduced GSH and oxidized GSSG (Owen and Butterfield, 2010). An essential antioxidant in cells is GSH. Through a two-step process, it is created from cysteine, glutamate, and glycine (Lu, 2013). In the initial stage, glutamate-cysteine ligase (GCL) joins glutamate and cysteine to create γ-glutamyl-cysteine. Next, catalyzed by glutathione synthetase (GSS), glycine is added to γ-glutamyl-cysteine to form GSH. Therefore, the ferroptosis inducer Erastin inhibits cysteine metabolism, preventing GSH synthesis, resulting in protein and membrane deterioration, lipid peroxide buildup, and eventually cell ferroptosis (Wang et al., 2020b).

GPX4 uses GSH as substrate to reduce membrane phospholipid hydroperoxide to harmless fatty alcohol (Forcina and Dixon, 2019). Therefore, GPX4 is considered a key antioxidant enzyme that inhibits ferroptosis by directly eliminating hydrogen peroxide in lipid bilayers and preventing the accumulation of lethal lipid ROS. GPX4 is closely related to the function of System Xc-. When System Xc-function is inhibited and GSH is depleted, GPX4 activity is indirectly suppressed. This leads to the oxidation of lipids by Fe^2+^ and the generation of ROS, thereby promoting ferroptosis (He et al., 2023).

### 3.3 Lipid peroxidation

Increasing evidence suggests that lipid peroxidation is a key feature of ferroptosis and a critical mediator in PD (Qiu et al., 2020). One of the primary constituents of cell membranes, lipids are essential for preserving the structural integrity of cells. The most prevalent oxidants in cells are reactive oxygen species (ROS). Because of their high polyunsaturated fatty acid (PUFA) content, cellular or organelle membranes are especially vulnerable to ROS damage. The ferroptosis phenotype caused by excessive lipid peroxidation is exacerbated by intracellular iron overload and GSH depletion (Rochette et al., 2022).

When iron overload occurs, a large amount of labile iron can participate in redox reactions. These redox reactions, catalyzed by the redox-active iron pool, are collectively referred to as the Fenton reaction (Reyhani et al., 2019). In the Fenton reaction, Fe^2+^ and H_2_O_2_ are oxidized to Fe^3+^ in a stoichiometric manner, generating OH^−^ and highly reactive ·OH. The Haber-Weiss reaction complements the Fenton reaction. Fe^3+^ interacts with ·O_2_^−^, reducing it to Fe^2+^ and producing O_2_. In the subsequent step, the Fenton reaction occurs, where Fe^2+^ reacts with H_2_O_2_ to produce ·OH, OH^−^, and Fe^3+^. Throughout this process, iron ions act as catalysts, facilitating the conversion of ·O_2_^−^ and H_2_O_2_ into ·OH, OH^−^, and O_2_. Although thermodynamically exergonic, this reaction demonstrates extremely low catalytic efficiency in solution, suggesting its contribution to superoxide toxicity is likely negligible (Koppenol and Hider, 2019). Subsequently, the antioxidant system is compromised, leading to the accumulation of lipid peroxides, which damages the cell membrane structure and ultimately results in cell death (Liu et al., 2022). GPX4 uses GSH as a co-substrate to reduce lipid peroxides to their corresponding alcohols and is a key regulator of intracellular lipid peroxides (Zhang et al., 2022). Inactivation of this enzyme leads to the accumulation of lipid peroxides and ferroptosis.

Malondialdehyde (MDA) and 4-hydroxynonenal (4-HNE) are highly reactive aldehydes produced during lipid peroxidation and are known to contribute significantly to cellular damage (Li et al., 2022a). These aldehydes can form adducts with proteins and DNA, leading to further cellular dysfunction and inflammation. In PD, elevated levels of MDA and 4-HNE have been observed, which correlate with increased oxidative stress and neuronal damage (Coles, et al., 2018). The accumulation of these aldehydes exacerbates the neuroinflammatory response, promoting the progression of neurodegeneration. Moreover, the presence of these aldehyde products can inhibit key cellular enzymes, including GPX4, thereby perpetuating lipid peroxidation and ferroptosis (Liu et al., 2021b).

In conclusion, abnormal iron metabolism, impaired antioxidant defense systems, and lipid peroxidation lead to ferroptosis, thereby accelerating the progression of PD. In recent years, iron chelators and antioxidants have attracted attention as potential therapeutic strategies, offering promising approaches to alleviate symptoms such as ptosis and slow the progression of PD (Lu, et al., 2023).

## 4 The connection between neuroinflammation and ferroptosis in PD

**FIGURE 3:**
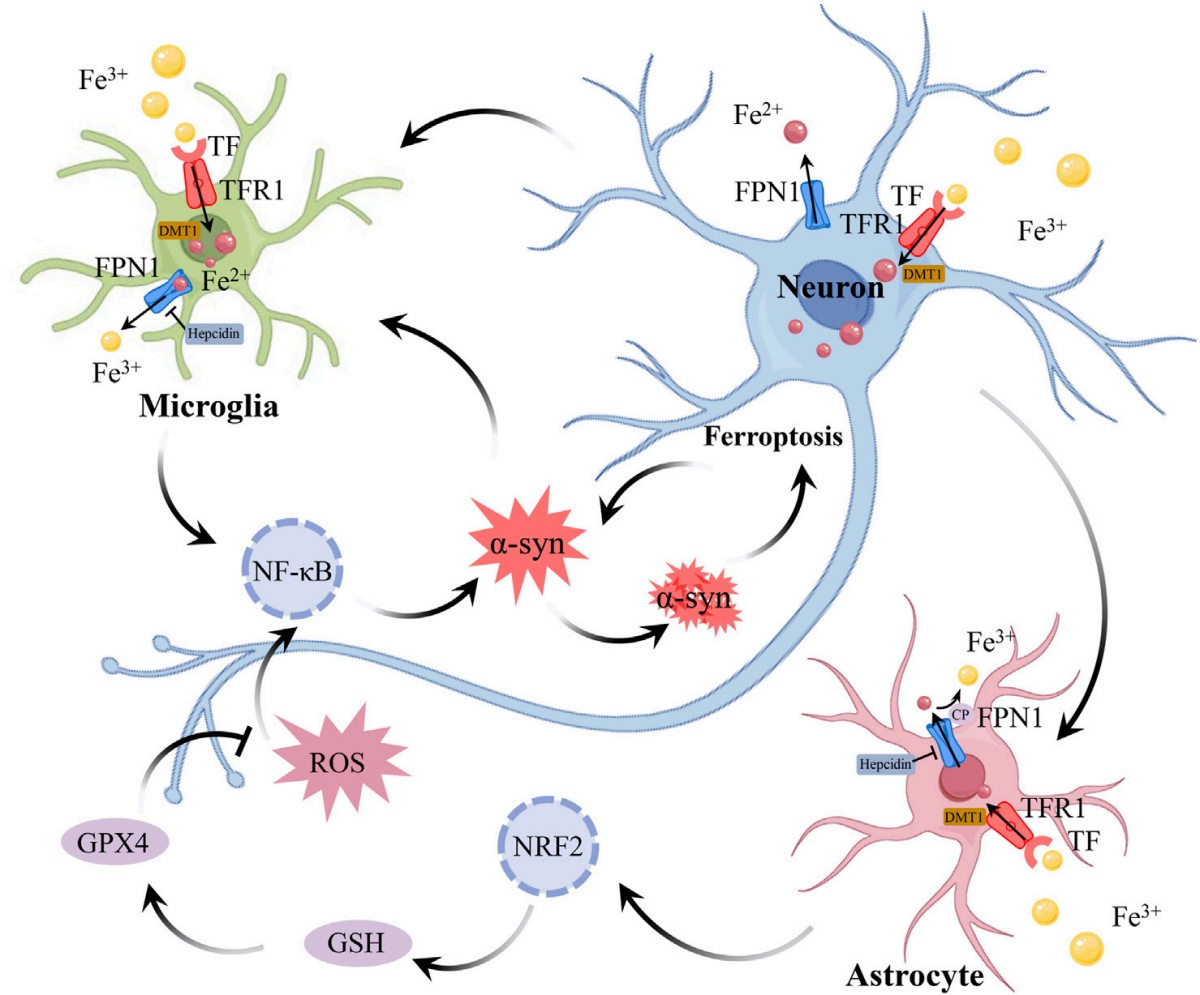
The connection between neuroinflammation and ferroptosis in PD. During neuroinflammation, the expression of DMT1 in neurons, astrocytes, and microglia induces the production of hepcidin in astrocytes and microglia, while downregulating the expression of FPN1 in all 3 cell types, leading to increased iron accumulation in neurons and microglia. Astrocytes absorb iron through DMT1, and regulate iron metabolism depending on the iron oxidase activity of CP. In addition, astrocytes can also regulate gene expression related to GSH by activating Nrf2 signaling pathway, thus reducing oxidative stress triggered by ROS. However, with the persistence of neuroinflammation, the protective function of astrocytes is gradually weakened, which leads to further injury of neurons. The pathological accumulation of α-syn is a key feature of PD. The accumulation of α-syn inhibits the transport of TF, leading to the accumulation of TF and an increase in intracellular iron levels. Iron can bind to α-syn and catalyze lipid peroxidation reactions, accelerating the oxidation and aggregation of α-syn, which in turn leads to further iron accumulation. In the case of iron deposition, intracellular oxidative stress activates NF-κB, which further aggravates neuroinflammation. The accumulation of iron leads to oxidative stress, which activates inflammatory signals, and neuroinflammation further destroys iron homeostasis and aggravates ferroptosis, which continuously aggravates the damage of DAn. The interaction between neuroinflammation and ferroptosis constitutes a vicious circle in the pathogenesis of PD. Abbreviations: DMT1, divalent metal transporter 1; FPN1, ferroportin 1; CP, ceruloplasmin; GSH, glutathione; NF-κB, Nuclear Factor Kappa-B; Nrf2, nuclear factor erythroid 2-related factor2; ROS, reactive oxygen species; TF, transferrin; DAn, dopaminergic neurons; PD, parkinson’s disease.

Neuroinflammation and ferroptosis are both critical events in the pathogenesis of PD (Fernández-Mendívil et al., 2021). On one hand, oxidative stress caused by ferroptosis exacerbates neuroinflammation (Li et al., 2016). On the other hand, the release of pro-inflammatory factors during neuroinflammation triggers ferroptosis in immune cells and neurons (Li et al., 2021b). Neuroinflammation and ferroptosis interact, acting as “partners in crime” to jointly promote dopaminergic neuron degeneration (Figure 3).

### 4.1 Neuroinflammation triggers dysregulation of iron homeostasis

Neuroinflammation and iron deposition are intertwined in the pathogenesis of PD, jointly driving cell death. In the SN of PD patients, activated microglia and reactive astrocytes have been found to release pro-inflammatory factors that induce neuronal death (Imamura et al., 2003; Braak et al., 2007). It has been reported that multiple signaling pathways, such as The Nuclear Factor Kappa-B (NF-κB), protein kinase B (Akt), and mitogen-activated protein kinases (MAPK), are involved in the inflammatory response of microglia and astrocytes, and interact with each other (Xu et al., 2018a; Wang et al., 2017a). Transcriptomic analysis has revealed a close association between various ferroptosis-related genes and PD-related genes (Jian et al., 2022). These changes reflect the involvement of neuroinflammation and iron deposition in the pathological progression of PD. In the rat PD model established with 6-hydroxydopamine (6-OHDA), a large number of iron-rich microglia were found in the damaged SN region (Olmedo-Díaz et al., 2017). In PD patient SN slices, a close relationship was found between iron accumulation and microglial proliferation (Martin-Bastida et al., 2021). Suggests that neuroinflammation and iron accumulation in PD may have an interactive relationship. Pathological accumulation of α-syn is a key feature of PD. The interaction between α-syn and components of the endomembrane system can inhibit TF transport, leading to the accumulation of TF and an increase in intracellular iron levels (McLeary et al., 2019). Additionally, α-syn oligomers suppress the lysosomal-mediated degradation of FTN (ferritinophagy), resulting in the disruption of FTN turnover and further contributing to iron accumulation (Xiao et al., 2018). Iron-bound α-syn catalyzes lipid peroxidation reactions, accelerating the oxidation and aggregation of α-syn, which in turn leads to further iron accumulation, creating a vicious cycle (Li et al., 2020). Microglia, as key players in neuroimmune responses, may also be involved in this process. The accumulation of iron in microglia may exacerbate their senescence-associated secretory phenotype, further intensifying neuroinflammation and the aggregation of α-syn (Guo et al., 2021). This further confirms the link between iron homeostasis dysregulation, neuroinflammation, and α-syn aggregation in PD.

Inflammatory stimulation upregulates DMT1 expression in neurons, astrocytes, and microglia, induces hepcidin production in astrocytes and microglia, and downregulates FPN1 expression in all 3 cell types, leading to increased iron accumulation in neurons and microglia (Urrutia et al., 2013). Pro-inflammatory cytokines (IL-6, IL-1β, TNF-α) released by microglia exacerbate neuronal iron deposition. These inflammatory factors increase neuronal iron accumulation by upregulating DMT1 and TFR1, and downregulating FRN1 expression (Wang et al., 2013). These inflammatory factors not only damage surrounding neurons but also activate the NF-κB and JNK signaling pathways in neurons, leading to increased expression of α-syn in neurons, which further exacerbates neurodegenerative changes (Angelova and Brown, 2018). Compared to LPS induction alone, co-induction with iron overload and LPS significantly increases the production of IL-1β, TNF-α, and ROS in microglia. This further amplifies the inflammatory response, promotes additional iron accumulation, and ultimately leads to neuronal death (Yauger et al., 2019). Astrocytes maintain iron homeostasis in CNS by regulating iron transport. They primarily absorb iron via DMT1 and rely on ceruloplasmin (CP) for its ferroxidase activity to oxidize Fe^2+^ to Fe^3+^, thereby facilitating iron export and inhibiting lipid peroxidation (Jeong and David, 2003). However, in the SN of PD patients, the ferroxidase activity of CP is reduced by approximately 80%, leading to increased iron accumulation and associated with neuroinflammation (Ayton et al., 2013). Additionally, astrocyte-released GDNF and brain-derived neurotrophic factor (BDNF) contribute to neuronal iron metabolism by lowering DMT1 expression, which in turn reduces iron buildup in neurons (Zhang et al., 2014). Additionally, due to the lack of a cystine transport system in neurons, their GSH synthesis relies on the xCT transporter in astrocytes and nuclear factor erythroid 2-related factor2 (Nrf2), which regulates the expression of GSH-related genes by activating the antioxidant response element (ARE) (Asanuma and Miyazaki, 2021). However, neuroinflammation causes neuronal damage and impairs the repair functions of glial cells. Therefore, iron accumulation and inflammatory factors work together to create a self-amplifying vicious cycle.

### 4.2 Ferroptosis metabolites activate inflammatory signaling

An important factor in neuroinflammation is iron. The disruption of the BBB allows neurotoxins such as iron to enter the brain, resulting in impaired function of neurons and glial cells (Riederer et al., 2023). Research indicates that the leakage of iron is associated with neurodegeneration and may exacerbate pathological conditions by inducing inflammatory responses and cellular damage (Al-Bachari et al., 2020).

During ferroptosis, the deficiency of GPX4 leads to elevated levels of intracellular ROS, triggering oxidative stress (Zheng and Conrad, 2020). ROS and oxidative stress are frequent characteristics linked to inflammation. NF-κB inflammatory signaling pathway can be triggered by ROS. NF-κB is also a redox-regulated transcription factor, primarily involved in the regulation of inflammation/immune responses, cell apoptosis, and cell growth (Karin et al., 2004). Since NF-κB is expressed in almost every type of cell, including microglia, neurons, and astrocytes, its functions in various tissues and cells may have a major influence on systemic inflammatory responses. The increase in ROS induces the dimerization of IKKγ/NEMO, thereby activating IκB kinase (IKK), which leads to the phosphorylation and degradation of the inhibitory IκB protein, transforming NF-κB from an inactive to an active state. After entering the nucleus, active NF-κB attaches itself to particular DNA sequences and controls the production of pro-inflammatory cytokines like TNF-α, IL-6, and IL-1β (Zhou et al., 2014). Prolonged accumulation of ROS and sustained activation of the NF-κB pathway may lead to a chronic inflammatory state, exacerbating tissue damage and the occurrence of various diseases.

Nrf2 is a key regulator of the constitutive or inducible molecular system that controls redox homeostasis, and its function may be inhibited in PD (da Costa Caiado et al., 2025). It has been reported that iron inhibits Nrf2 promoting α-syn aggregation, which ultimately exacerbates ferroptosis (Gan and Johnson, 2014). Consistent with this finding, overexpression of Nrf2 has been shown to reduce the formation of α-syn aggregates in the CNS (He et al., 2013). The two opposing pathways, Nrf2 and NF-κB, can interfere with each other at the transcriptional level. Specifically, Nrf2 inhibits the activation of the NF-κB pathway by enhancing antioxidant defenses, thereby reducing ROS-mediated inflammatory responses (Soares et al., 2004). In addition, Nrf2 prevents the degradation of IκB, thereby blocking NF-κB nuclear translocation and the transcription of pro-inflammatory genes. In contrast, NF-κB can inhibit Nrf2 activity by enhancing the recruitment of histone deacetylase 3 (HDAC3) to the ARE region, thereby preventing the transcription of ARE genes (Wakabayashi et al., 2010). In summary, the interplay between the Nrf2 and NF-κB pathways highlights the critical balance between oxidative stress and inflammation.

Lipid peroxidation is a significant marker of ferroptosis. Lipids, as the main components of biological membranes, are prone to oxidative modification and serve as primary targets for ROS (Spickett, 2020). During lipid peroxidation, PUFAs, such as ω-6 fatty acid (AA), are oxidized, resulting in the formation of lipid free radicals and hydroperoxides. These products not only interact with DNA and proteins, altering membrane integrity and signal transduction, but also affect the expression of antioxidant and inflammatory genes such as Nrf2 and NF-κB. Epoxy fatty acids, leukotrienes, and prostaglandins are examples of bioactive eicosanoids that are produced by AA using lipoxygenases, which include cytochrome P450 enzymes, LOX, and cyclooxygenases (COX). Under normal circumstances, COX-2 controls the nervous system’s synaptic and cerebral vascular plasticity. However, when lipid peroxidation occurs, COX-2 overexpression causes neurotoxicity via prostaglandin E2, which results in secondary neuroinflammatory injury. Pro-inflammatory cytokines including TNF-α and IL-1β have been shown to decrease in concentration when COX-2 inhibitors are used (Lee et al., 2005). Additionally, the injection of heme into the brain leads to lipid peroxidation, which not only damages cell membranes but also activates the NLRP3 inflammasome through the production of toxic lipid oxidation products. This process promotes the release of inflammatory factors such as IL-1β, further exacerbating neuroinflammation (Vasconcellos et al., 2021).

## 5 Potential therapeutic strategies

**FIGURE 4:**
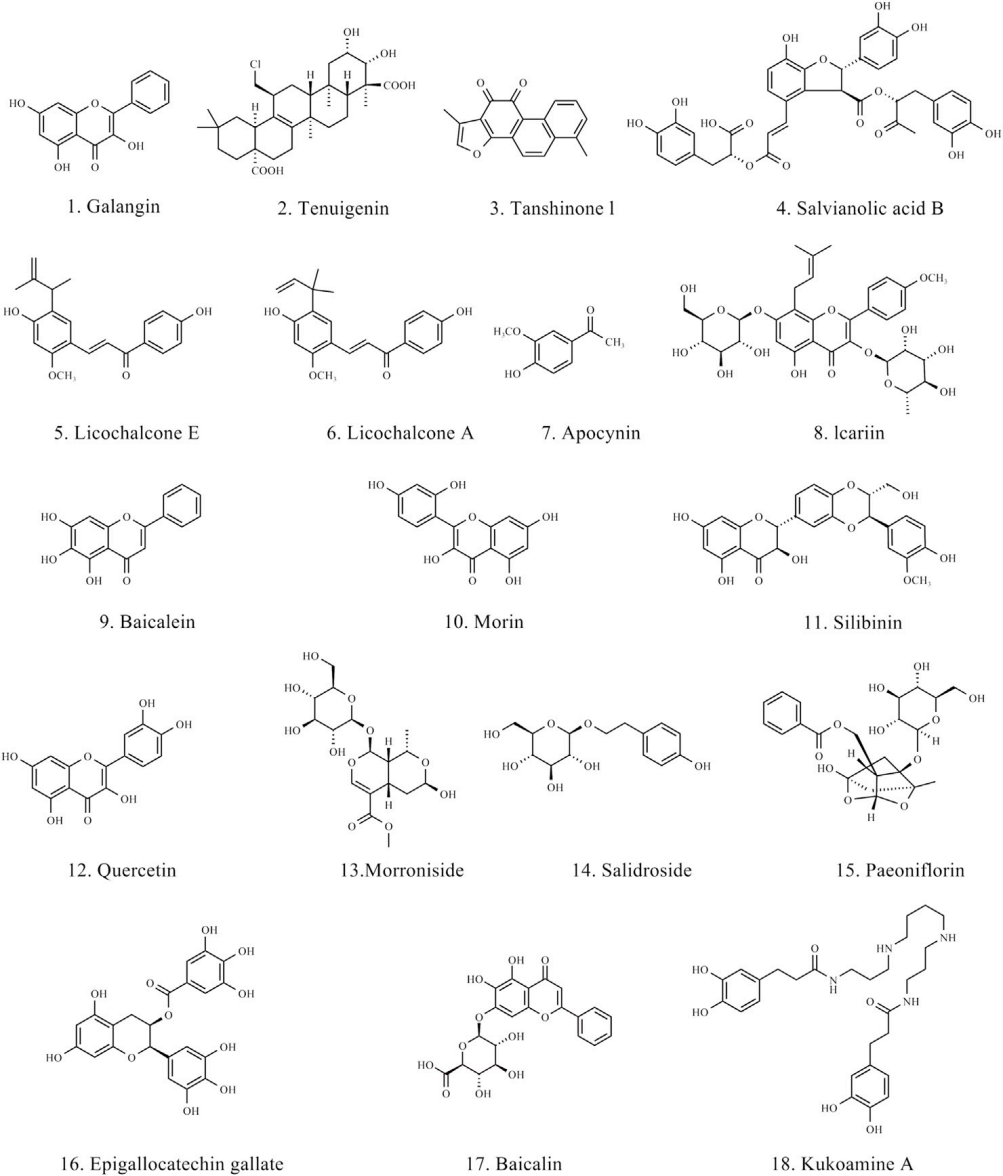
Chemical structure of natural products for treating PD.

In the therapeutic strategies for PD, natural products have shown great potential, particularly in modulating neuroinflammation and ferroptosis. Various natural products have been proven to inhibit the pro-inflammatory responses of microglia and astrocytes, promoting their transformation into an anti-inflammatory phenotype, thereby reducing neuronal damage. Moreover, natural products inhibit ferroptosis by improving oxidative stress and regulating iron metabolism. The chemical structures of the natural products are shown in Figure 4. The BBB permeant of all the natural products in the table is based on predicted results obtained from SwissADME.

### 5.1 Natural products that modulate microglial

The rhizomes of the *Alpinia officinarum* Hance (Zingiberaceae) contain a natural flavonol called galangin, which has long been used to treat gastrointestinal disorders. Research has demonstrated that galangin has antibacterial, anti-inflammatory, and antioxidant qualities and is a recognized PPARγ activator (Choi et al., 2017). According to reports, galangin reduces inflammation associated with the JNK, Akt, and NF-κB pathways by activating the Nrf2/CREB signaling pathway and inhibiting M1 activation in LPS-stimulated BV-2 cells. Tenuigenin is a natural terpenoid compound found in the roots of the *Polygala tenuifolia* Willd. (Polygalaceae), and has long been used to treat cognitive impairment, neurasthenia, insomnia, and other conditions. Studies have shown that tenuigenin exhibits neuroprotective effects, including antioxidant, anti-aging, and anti-inflammatory properties (Wang et al., 2017b). By triggering the Nrf2/HO-1 pathway, tenuigenin decreases LPS-induced microglial M1 activation in a dose-dependent manner. In a PD animal model, tenuigenin significantly improved motor behavior and reduced dopaminergic neuronal damage by inhibiting the NLRP3 inflammasome (Fan et al., 2017). Studies have demonstrated the neuroprotective properties of salvianolic acid B (SAB) and tanshinone I (TSI) from *Salvia miltiorrhiza* Bunge (Labiatae) against PD. By blocking NF-κB nuclear translocation, TSI can lessen LPS-induced microglial M1 activation while maintaining the expression of certain M2 markers (Wang et al., 2015). SAB, by activating the Nrf2 signaling pathway, shifted microglia from the M1 phenotype to the M2 phenotype, reducing neuronal damage (Zhang et al., 2017). The active ingredients licochalcone E (LicoE) and licochalcone A (LicoA), which have anti-inflammatory qualities, were isolated from the roots and rhizomes of *Glycyrrhiza glabra* L. or G. *inflata* Batal. (Leguminosae). Studies have shown that LicoE prevents dopaminergic neuronal degeneration by upregulating the Nrf2/ARE pathway and inhibiting M1 microglia-induced inflammatory responses (Kim et al., 2012). By blocking the ERK 1/2 and NF-κB pathways, LicoA lowers the production of pro-inflammatory markers in BV-2 cells. In an animal model of PD, it also reduces microglial M1 activation and neuronal death, which ameliorates PD symptoms (Huang et al., 2017). Apocynin is a bioactive phenol extracted from the roots of *Apocynum venetum* L., and as a NOX2 inhibitor, it can prevent ROS production and reduce inflammatory responses (Philippens et al., 2013). Another study also confirmed that Apocynin, by targeting NADPH oxidase, can inhibit M1 microglial activation and reduce the production of cytokines, ROS, and inflammatory factors (Sharma and Nehru, 2016). Icariin is extracted from Epimedium Linn. and possesses various bioactivities, including antioxidant and anti-apoptotic effects. According to studies, icariin improves motor function, lessens microglial M1 activation and neuronal death, and inhibits the JNK, p38 MAPK, and NF-κB pathways to minimize the generation of ROS and inflammatory cytokines brought on by LPS (Zeng, et al., 2010) (Table 1).

**TABLE 1:** Natural products that modulate microglial.

Natural products	BBB permeant	Herb	Application model	References
Galangin	NO	*Alpinia officinarum* Hance	LPS injected male ICR mice LPS induced BV-2 cells	Choi et al. (2017)
Tenuigenin	NO	*Polygala tenuifolia* Willd	LPS injected male ICR mice MPTP injected male C57BL/6 mice LPS induced BV-2 cells ATP or MSU induced BV-2 cells	Wang et al., 2017a; Fan et al., 2017
Tanshinone I	YES	*Salvia miltiorrhiza* Bunge	MPTP injected male C57BL/6 mice LPS induced BV-2 cells	Wang et al. (2015)
Salvianolic acid B	NO	*Salvia miltiorrhiza* Bunge	LPS induced primary microglia	Zhang et al. (2017)
Licochalcone E	YES	*Glycyrrhiza glabra* L. or *G. inflata* Batal	MPTP injected male C57BL/6 mice LPS induced BV-2 cells 6-OHDA induced SH-SY5Y cells	Kim et al. (2012)
Licochalcone A	YES	*Glycyrrhiza glabra* L. or *G. inflata* Batal	LPS injected wistar rats LPS injected wistar rats	Huang et al. (2017)
Apocynin	YES	*Apocynum venetum* L	MPTP induced marmoset monkeys LPS induced SD rats	Philippens et al., 2013; Sharma and Nehru, 2016
Icariin	NO	Epimedium Linn	LPS induced primary microglia	Zeng et al. (2010)

### 5.2 Natural products that modulate astrocytes

Baicalein is a flavonoid compound isolated from the roots of *Scutellaria baicalensis* Georgi, showing broad pharmacological activity, particularly in anti-inflammatory and neuroprotective effects. Research has demonstrated that baicalein suppresses the NF-κB signaling pathway in MPTP-induced PD mice models, which lowers the production of pro-inflammatory cytokines and, in turn, decreases astrocyte activation (Lee et al., 2014). Astrocyte activation is a common pathological process in PD, closely associated with motor dysfunction. By suppressing this inflammatory response, baicalein effectively alleviated PD-related motor impairments, demonstrating its potential as a neuroprotective agent. Additionally, morin, a natural flavonoid primarily found in the leaves of *Maclura pomifera* (Raf.) C. K. Schneid., has also demonstrated regulatory effects on neuroinflammation. It has been reported that morin can reduce astrocyte activation, inhibit inflammatory responses, and subsequently prevent the degeneration of DAn in an MPTP-induced mouse model of PD (Lee et al., 2016). Silibinin, the main active component extracted from *Silybum marianum* (L.) Gaertn., has various bioactivities, including anti-inflammatory and antioxidant effects. In PD research, silibinin has also shown significant neuroprotective effects. Studies have found that silibinin can significantly alleviate motor deficits in MPTP-induced PD mouse models, reduce dopaminergic neuronal death, and inhibit excessive astrocyte activation by inhibiting the ERK and JNK signaling pathways as well as the expression of cyclooxygenase (COX) (Lee, et al., 2015) (Table 2).

**TABLE 2:** Natural products that modulate astrocytes.

Natural products	BBB permeant	Herb	Application model	References
Baicalein	NO	*Scutellaria baicalensis* Georgi	MPTP injected male C57BL/6 mice MPP + induced primary neuron and primary astrocyte	Lee et al. (2014)
Morin	NO	*Maclura pomifera* (Raf.) C. K. Schneid	MPTP injected male C57BL/6 mice MPP + induced primary neuron and primary astrocyte	Lee et al. (2016)
Silibinin	NO	*Silybum marianum* (L.) Gaertn	MPTP injected male C57BL/6 mice MPP + induced primary astrocyte	Lee et al. (2015)

### 5.3 Natural products that regulate ferroptosis

The Nrf2/GPX4 pathway plays a crucial role in the defense against oxidative stress and ferroptosis. Quercetin is a natural flavonoid, which is widely found in various vegetables, fruits and Chinese herbal medicines. It is reported that quercetin can prevent ferroptosis by improving mitochondrial function and reducing mitochondrial ROS (Lin et al., 2022). This study pointed out that quercetin’s inhibitory effect on ferroptosis depends on Nrf2, and the protein levels of GPX4 and SLC7A11 are regulated by Nrf2 *in vitro* and *in vivo*. In addition, it has been found that quercetin can counteract lipid peroxidation through the Nrf2/GPX4 pathway, thereby inhibiting ferroptosis (Jiang et al., 2023). Network pharmacology analysis reveals that two potential targets of quercetin in the treatment of PD are the dopamine receptor D2 (DRD2) and dopamine receptor D4 (DRD4). Activation of these receptors can regulate dopaminergic signaling and may help alleviate motor symptoms in PD. GO and KEGG analysis indicate that the therapeutic effects of quercetin on PD primarily involve G-protein coupled receptor signaling pathway, negative regulation of voltage-gated calcium channel activity, and positive regulation of dopamine uptake involved in synaptic transmission (Lakshmi et al., 2023). Morroniside is the active ingredient in the traditional Chinese medicine *Cornus officinalis* Sieb. *Et Zucc*. Morroniside demonstrated neuroprotective effects in PD mice models induced by MPTP, activating the Nrf2, upregulating GPX4 expression, promoting antioxidation, and inhibiting ferroptosis, which protected dopaminergic neurons from oxidative damage (Li et al., 2023). Salidroside is a major component extracted from *Rhodiola rosea* L. It has been reported that Salidroside exerts significant anti-ferroptosis effects by upregulating GPX4, inhibiting ROS levels, and reducing the production of lipid peroxides. However, this protective effect is significantly weakened upon treatment with the Nrf2 inhibitor (ML385), further validating the crucial role of the Nrf2/GPX4 pathway in Salidroside’s anti-ferroptosis action (Wu et al., 2017; Shen et al., 2024). Paeoniflorin is a water-soluble monoterpenoid glycoside extracted from the root of *Paeonia lactiflora* Pall. It has been reported that paeoniflorin exerts protective effects on primary DAn by activating Nrf2 to promote GPX4 expression and inhibit ferroptosis, with the anti-ferroptosis role of the Nrf2/GPX4 pathway further validated through ML358 (Wang et al., 2022b).

Regulating iron metabolism is another important strategy for inhibiting ferroptosis. Epigallocatechin gallate (EGCG) is a polyphenol with antioxidant, anti-inflammatory and iron chelating properties extracted from green tea. The research shows that EGCG regulates the iron export protein iron transporter in SN, reduces oxidative stress, and plays a neuroprotective role in MPTP-induced functional and neurochemical defects in mice (Xu et al., 2017). Furthermore, studies have shown that EGCG can simultaneously prevent neuronal apoptosis induced by TNFα and H_2_O_2_. This neuroprotection may be mainly mediated by hepcidin and partly by ferroportin, which plays a role in inhibiting oxidative stress and inflammation (Xu et al., 2018a). Baicalin is one of the active components of *S. baicalensis* Georgi. Iron is not only accumulated in the SN, but also in the striatum of globus pallidus, granular layer of dentate gyrus of hippocampus, dentate muscle-socket body and facial nucleus of cerebellum. Baicalin can significantly inhibit iron deposition in these brain regions and significantly reduce the loss of tyrosine hydroxylase positive cells (Xiong et al., 2012). On this basis, it has been reported that baicalin can reduce the accumulation of iron in SN and has the mechanism of inhibiting iron accumulation in PD rats (Guo et al., 2014). Kukoamine A is the effective active ingredient of dried root bark of *Lycium barbarum* L. Kukoamine A can chelate the iron content in cells and reduce the inflow of iron, thus maintaining the iron homeostasis of cells and avoiding neuronal death caused by iron deposition in cells (Li et al., 2021c) (Table 3).

**TABLE 3:** Effective herbal extract for relieving PD.

Natural products	BBB permeant	Herb	Application model	References
Quercetin	NO	Various herbs	MPTP injected male C57BL/6 mice Rotenone induced male rats MPP + induced PC12 cells, SH-SY5Y cells and primary neurons	Lin et al., 2022; Jiang et al., 2023; Lakshmi et al., 2023
Morroniside	NO	*Cornus officinalis* Sieb. Et *Zucc*	MPTP injected male C57BL/6 mice MPP + induced PC12 cells	Li et al. (2023)
Salidroside	NO	*Rhodiola rosea* L	MPTP injected male C57BL/6 mice Erastin induced SH-SY5Y cells MPP + induced SH-SY5Y cells	Wu et al., 2017; Shen et al., 2024
Paeoniflorin	NO	*Paeonia lactiflora* Pall	MPP + induced primary neurons	Wang et al. (2022a)
Epigallocatechin gallate	NO	Green tea	MPTP injected male C57BL/6 mice TNFα or H_2_O_2_ induced N27 cells	Xu et al., 2017; Xu et al., 2018b
Baicalin	NO	*Scutellaria baicalensis* Georgi	Rotenone induced male Wistar rats Ferric ammonium citrate induced C6 cells	Xiong et al., 2012; Guo et al., 2014
Kukoamine A	NO	*Lycium barbarum* L	6-OHDA induced PC12 cells	Li et al. (2021a)

## 6 Conclusion

PD is a multifactorial neurodegenerative disorder, with neuroinflammation and ferroptosis playing critical roles in its pathogenesis. The intricate interplay between neuroinflammation, driven by activated microglia and astrocytes, and iron-dependent oxidative stress marks a key convergence point that accelerates dopaminergic neuronal loss. Therapeutic strategies targeting these pathways hold promise for altering disease progression.

Natural compounds derived from traditional medicines have shown efficacy in alleviating neuroinflammation and ferroptosis. These compounds exert neuroprotective effects by modulating key signaling pathways, such as NF-κB and Nrf2, which are central to controlling inflammation and oxidative stress. Preclinical studies have highlighted the potential of these agents to reduce neuronal damage, offering a promising avenue for future therapeutic interventions. However, we acknowledge that much of the data discussed relies on animal model studies, which may not fully replicate human physiology. Additionally, due to the multi-target nature of natural products, there is a possibility of off-target effects, which could lead to unintended interactions or side effects.

Looking ahead, there is a pressing need to further explore the molecular mechanisms underlying the link between neuroinflammation and ferroptosis in PD. Given that natural products often encounter challenges, such as poor BBB permeability, future research should focus on optimizing drug delivery systems, enhancing the chemical modification of these compounds, refining targeted therapy strategies, and precisely controlling drug release. Clinical studies are also needed to evaluate the safety, efficacy, pharmacokinetics, and potential side effects of these natural compounds in human subjects. As our understanding of the interconnected processes in PD advances, new combination therapies may emerge that target both the inflammatory and oxidative components of the disease. Such strategies could lead to innovative treatments that not only alleviate symptoms but also slow disease progression, offering hope for better outcomes in PD patients.
